# Do we really need financial literacy to access the behavioral dynamics of generation Z? A case of Oman

**DOI:** 10.1016/j.heliyon.2024.e32739

**Published:** 2024-06-24

**Authors:** Mohammad Shahfaraz Khan, Imran Azad, Sanyo Moosa, Mohd Yousuf Javed

**Affiliations:** a College of Economics and Business Administration, University of Technology and Applied Sciences-Salalah (Oman), Oman; b Faculty Department of Business Administration, Aligarh Muslim, Kishanganj Centre, Bihar, India

**Keywords:** FL, Financial literacy, FB, Financial behavior, SEM, Compulsive buying

## Abstract

The ability to manage money has been identified as a vital talent. In this context, financial literacy has a role to play. There are significant gaps, though, such as the financial connection to human behavior. The purpose of this study is to look at the overall effects of financial literacy (FL) on the behavioural aspects of materialism (ML), compulsive buying (CB), and tendency to debt (TD). It attempts to develop and evaluate a model that is based on these variables and to examine the relationships between these research constructs. The study looked at 233 respondents mainly students studying in Omani universities.

A comprehensive literature review, confirmatory factorial analysis, Structural Equation Modelling (SEM), and comprehensive analysis were used to develop research hypotheses. The primary findings suggested that financial literacy had the most significant impact on compulsive purchasing behaviour in comparison to materialism and the propensity to accumulate debt, among the direct relationships that were suggested. The study's findings have important implications for the formulation of public policy and other interested parties, as financial literacy is advantageous for individuals with poor financial health and facilitates the participation in other psychological behaviours.

## 1 Introduction

Students at universities contribute significantly to the development of a nation as educated youth. They are in a stage of development where they will eventually become much more financially independent [1]. highlighted the dynamics of financial literacy among Generation Z and concluded that financial literacy affect the self-efficacy among GenZ. They must manage their finances and participate in the nation's development. The knowledge and skills students acquire while attending university last a lifetime and aid them in making sensible decisions. Furthermore [2] the financial literacy (FL) has a huge impact on the Gen Z and Gen X investment behavioral dynamics. Similar to this, acquiring financial knowledge and abilities enables people to improve their financial well-being and determine whether they will flourish or suffer financially. The ability to develop and make wise financial judgments requires financial literacy. People must be able to fully capitalise on the opportunities presented by the financial products and services that are available to society, as they are intricate. Nevertheless, it is equally important for individuals to be cognizant of the risks and uncertainties that accompany their decisions. As a consequence, financial literacy has become indispensable for complete social integration [3]. Furthermore [4], posits that in the twenty-first century, increasing financial literacy is essential for fostering economic development in any global economy. Five The responses from 1479 respondents were used to analyze the personality traits and gender in relation to wagering. One of the aspects of over-indebtedness has been examined, and the findings demonstrate that women are more likely to be over-indebted [5]. Utilized data from 2009 to 2018 to examine the correlation between FB (financial behaviour) and FK (financial knowledge). The findings indicated a positive association between FB and FK, and robustness was also verified through the use of multiple regressions (see Exhibit 01, Exhibit 02, Exhibit 03, Exhibit 04).

**Exhibit 01:**
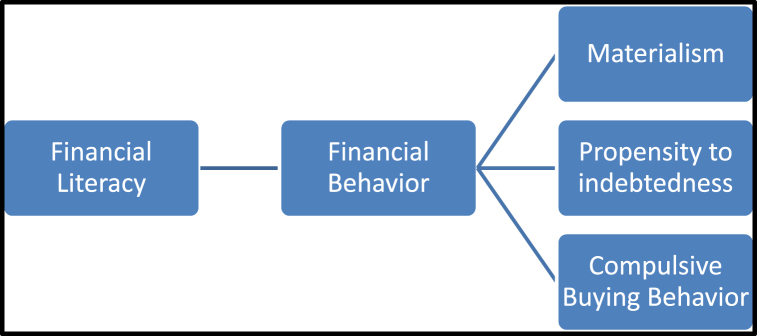
Showing conceptual model.

(Prepared by Authors)

**Exhibit 02:**
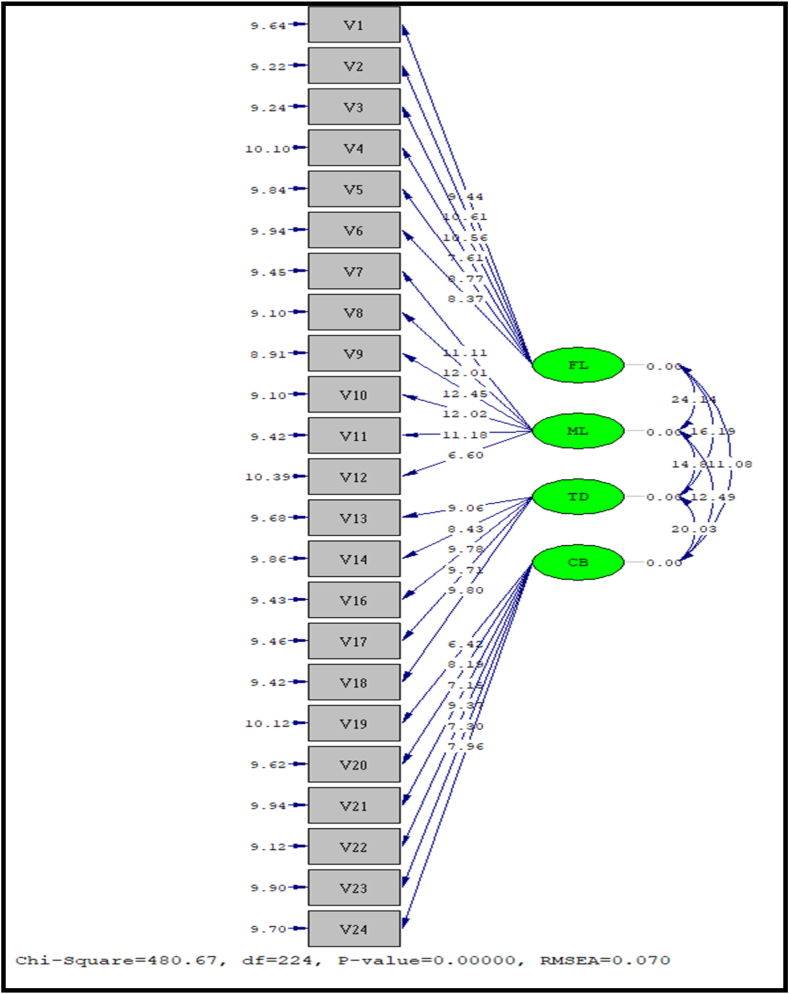
Showing CFA for all study scales.

**Exhibit 03:**
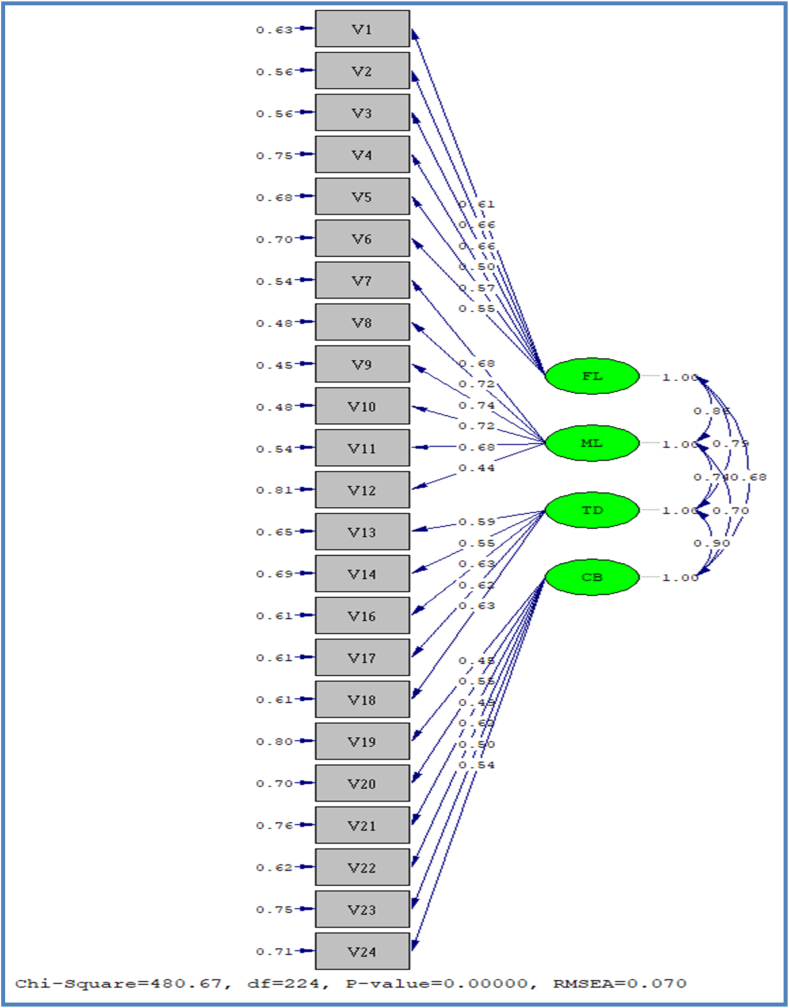
Showing t values for all the study scales.

**Exhibit 04:**
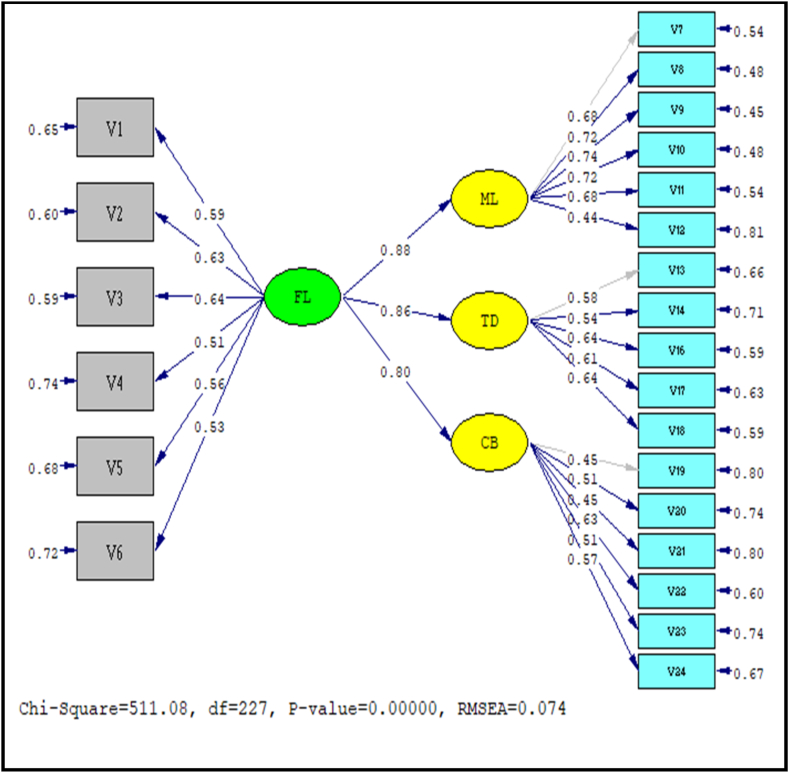
Showing structural model for all study scales.

The term "financial literacy" denotes the possession of the minimal minimum of FK (financial knowledge) and insights needed to make wise decisions in financial matters. The individuals' decision-making procedures, additionally their macroeconomic and microeconomic activities, are influenced by the application of this knowledge and information. Seven Concentrated on the learning patterns of Generation Z during the COVID-19 pandemic. Our research focused on Generation Z, as the majority of respondents were members of this demographic. In the broadest sense, financial literacy is the knowledge that can be employed to make decisions and participate in economic activities, such as savings, investments, and consumption. Age, gender, major or area of study, education, and family history are among the factors that will be assessed in the proposed study, which is the first of its kind to assess the degree of financial understanding amongst UTAS students. Eight A questionnaire study was employed to examine the socioeconomic elements and cultural implications of FL on Generation Z in Vietnam. The results of the study suggested that respondents with high incomes better handle their financial affairs. It is crucial to develop models that can ascertain the impact of FL on financial behaviour and choices to recognize the significance and potential consequences of implementing nationwide initiatives to promote FL. Financial advisors could benefit from the use of these models to develop products that are customized to fulfill the demands of a wide variety of customers. Furthermore, any confirmation of the impact of FL on materialism, habitual purchasing behaviours, and the tendency to accumulate debt may be employed to create remedies from a behavioural perspective.

This study shall open some important insights in the light of FL and FB. It is taken up to explore the impact of FL on financial behavior (FB) especially on Omani students who are the part of Generation Z. Furthermore, the research idea is to explain the degree of FL among UTAS students from Oman. To investigate the FL levels of students with other financial behavior constructs such as materialism and propensity to get indebted in students with various socio-demographic profiles.

## 2 Literature review

There is currently no complete model for estimating the degree to which financial literacy, when paired with other behavioral characteristics, can raise an individual's likelihood of falling into debt. The literature on this topic contends that financial literacy is a complex notion. This implies that this might have an even bigger impact than it already does when examined separately. By attempting to create a model to examine the holistic impact of financial literacy on behavioral qualities including materialism, compulsive buying, and a tendency towards debt, this study innovates.

In order to accomplish this, it is recommended that a structural equations model be constructed and validated in order to simultaneously estimate the number of direct or indirect dependency relations [6]. Financial stability, monetary policy, and financial inclusion were examined using data spanning from 2004 to 2020. Financial inclusion and stability were found to be directly correlated by the results.

Nevertheless, despite its importance, many studies have shown that the majority of the global population still lacks financial literacy, underscoring the necessity of taking action to resolve this matter ([3,4]). Even at the global governance level, there are currently issues with financial literacy.

[7] The significance of financial literacy and inclusion at an early stage has been discussed in this paper. The SFEM (social financial education model) has been used and validated in this study at an early stage. Results indicated that children, especially those in Generation Z, might become financially stable by media in the FSE (financial social education) paradigm.

Financial literacy is described by the “Organization for Economic Co-operation and Development (OECD)” [3] as “a collection of the diligence, knowledge, skills, attitudes, and behaviors required to make wise financial decisions and ultimately achieve individual financial well-being”. Furthermore, according to Ref. [8], financial literacy includes two components: comprehension, which refers to a person's financial awareness and is also known as financial edification, and use, which is the application of that information to individual money management. As a result, even if someone knows finances, they must also have the abilities and self-assurance to put that information to use when making decisions to be deemed financially literate [8].

Interest rates are high, exceptionally in Oman where many people make use of credit cards, and the government frequently promotes consumption-based growth. In November 2017, the proportion of debt-ridden Omani families reached 32.2 % (CNC, 2017) [9]. studied the household food and financial literacy by using ETR the results were drawn that FL is positively correlated with households’ literacy.

[10] Investigated the predicting ability of demographic data and the big five personality traits in stock trading. Results indicated that more neurotic and open investors make more money in the market. Social traits have a positive impact on stock trading. In this context, it becomes imperative to understand the direct and indirect effects of financial literacy on the propensity for indebtedness in order to develop national financial literacy strategies for the definition of credit policies by the various financial agents and the management of non-payment.

[11] Explored the dimensions of the project and investor's related aspects in Indonesia on investor's intention. Findings suggested that return parameters positively affect the investor's willingness to change the investment projects.

[12] examined how behavioral, psychological, and demographic characteristics affect the way people make financial decisions; using primary data from 634 investors the findings were drawn. SEM has been used to analyze the data. The results indicated the importance of both spontaneous financial decision-making and FL.

### 2.1 Research gaps

Although this subject has been extensively investigated, this study chooses a unique group of respondents and setting, making it a critically important study regarding Oman's student population's financial behavior and understanding of finance. Following are a few justifications for this study.

1. According to a review of the current literature, although financial literacy has been the subject of many inquiries lately, not much study has been done on the topic of RT mission units. However, there hasn't been much research done on evaluating FL within the framework of Omani students. Therefore, the goal of this work is to fill this knowledge research gap.
2. There is a paucity of research on financial literacy in the country of Oman, compared to the large number of studies conducted in the Western market. Understanding the dynamics of FL in this quickly changing Omani context is crucial because of the growing materialism and commercialization in the Omani context.
3. Since the absence of financial knowledge may endanger the financial stability of a household and raise the possibility of debt, the authors contend that financial competencies are a matter of concern. Consequently, this research is done.
4. Most of the studies on this subject are theoretical investigations since a large portion of the studies in this area are founded on literature surveys, secondary data sources, and theoretical advancement. Qualitative evaluation using original sources for data acquisition.
has been used to assess these traits empirically and generalize the findings more frequently than empirical measurement.
5. As expected, there was not a single study in the literature that addressed the financial behavior of the youth Gen z. Therefore, by carrying out the study there, it is possible to fill this knowledge gap.
6. Several studies have focused on the industrial and service sectors but university students have gotten far less attention. Given the urgency of the issue, research into these characteristics in this context is crucial.
7. Given the foregoing, this study is exceptional and cutting-edge in that it assesses the real direct effects of FL on other behavioral traits that have previously been examined separately.

### 2.2 Sample frame

Only students studying at UTAS-Oman are the subject of this study. The main population of interest for this proposed study is the graduating class of university students. University students' financial literacy levels can be evaluated to reveal how capable the young population is in terms of financial decisions making and evaluating risks. The study's findings will aid in the formulation of plans of action for both educators and policymakers who are fostering the growth of entrepreneurial activity.

### 2.3 Development of theoretical frame

#### 2.3.1 Financial literacy: definition

No single definition of financial literacy (FL) exists to date. FL is described as having the "knowledge and understanding of financial concepts and risks, and the skills, motivation, and confidence to apply such knowledge and understanding to make effective decisions across a range of financial contexts, to improve one's own and society's financial well-being, and to enable participation in economic life," in another definition the capacity to apply information and abilities to handle finances is referred to as having FL (financial literacy) [13]. FL is described as "the knowledge of fundamental financial concepts and capacity to perform simple computations" [13],. Financial literacy was defined by Ref. [8] as both possessing and applying knowledge about personal finance. “It is the capacity to use information and skills to manage financial resources successfully for a lifetime of financial well-being”, according to the U.S. (Financial Literacy and Education) Commission (2007) [14].

The word "financial literacy" lacks a singular, straightforward definition as it is a complicated subject and many includes different elements, beginning with the comprehension of fundamental concepts. Financial concepts are being explored along with the capability and self-assurance needed to effectively handle one's funds to ultimately develop efficient conduct.

Individuals consistently make decisions during their lives, encompassing many aspects such as financial decision-making. People must have the knowledge and abilities to handle their finances well in order to make wise financial decisions [15]. An individual's degree of success is largely determined by their ability to make wise financial decisions [16].

FL is a term that describes a person's degree of financial concept understanding, knowledge, and perception [17]. Financial literacy encompasses the comprehension and application of knowledge related to the management of financial resources. The term may also be characterized as the capacity to exercise discernment and reach conclusions about the management of financial resources [18]. The words "financial knowledge" and "financial literacy" have been employed in writings similarly [19]. The definition of financial literacy has transformed, encompassing five distinct categories. These categories include an understanding of financial concepts, effective communication skills related to financial concepts, recognition of the significance of individual money management, the ability to make wise financial choices, and assurance in meeting financial needs through well-thought-out plans [20].

The utilization of financial management information serves as an evaluative tool [21]. The research focus revolves around financial knowledge, specifically examining two aspects. The first aspect is people's capacity to use and apply general financial information in an efficient manner. The second part is estimating financial literacy by using particular financial understanding as a stand-in Ref. [22]. [17] Reported a study that assessed FL through the examination of general FL, financial planning, investments and knowledge with taxation. Several other scholars have also devised a knowledge scale for assessing financial literacy, encompassing several competencies such as deposit management, debit transactions, insurance policies, investment strategies, and general financial knowledge. Additionally, this scale may also incorporate individuals' self-assessment of their financial acumen [23,24].

The OECD's definition of financial literacy, which combines conscientiousness and knowledge, stands out for its representativeness among the many definitions and dimensions of the concept (OECD, 2015) [4]. Assert that FL causes consumers to make effective conclusions in light of the vast array of financial goods available. As a result, they are more likely to become indebted [25]. As a result, one of the research hypotheses that can be supported is the one that links financial literacy and propensity for debt.

[26] Conducted a study on how financial literacy and excessive debt are related. Only one out of three of the respondents were successful in using financial theories to comprehend day to day financial choices, like to know how of credit card's function, which indicated low degrees of financial education. As a result, these individuals would presumably bargain at great expense and with increased lending fees. Since the absence of these abilities may endanger the financial stability of a household and raise the possibility of debt, the authors contend that financial competencies are a matter of concern. People who cannot distinguish between a safe investment and one that might be quite dangerous are more likely to be vulnerable to the variety of available monetary products on the market and will experience financial issues like debt and default [27].

#### 2.3.2 Financial literacy: significance

Economic education is regarded as the cornerstone of financial literacy when it comes to subjects like savings, consumption, consumer choice (risk aversion, discount rates), economic environment (investment hazards), social security, etc. [28] It takes into account a wide range of factors, including spending and saving habits, individual finance, value estimation, taxes, consumer credit reports, insurance premiums, contracts for deposit accounts, loans, collateral, use of credit cards, insurance-related issues, health insurance, insurance contracts, pension funds, mutual funds and returns, risky investments, interest rate dynamics, and bond prices are some of the concerns associated with asset liquidity.

According to Ref. [29] financial literacy is a crucial component of everyone's existence. In terms of financial literacy, it was discovered that college students performed better than a typical sample of people. Additionally, just as local doctors offer advice to their communities, students pursuing higher education could provide financial expertise to their community.

The majority of FL studies highlight decisions, savings and investing behavior, and financial literacy as the drivers. A concern in both industrialized and developing countries is financial illiteracy, and studies have shown that it is particularly prevalent in low-income nations. A variety of populations have been the target of earlier financial literacy studies, including the young, young professionals, retired people, investors, older investors, high school students, individuals, and university students. Different demographic characteristics, such as class, major study areas, family education, and family income, are studied concerning university students. Families are crucial for financial literacy because they are the main source of financial knowledge for university students. According to Ref. [30], there are differences in FL-levels among university students grounded on age, class, and major streams of study. Family and education are the primary sources of information. The majority of financial knowledge regarding spending and saving habits comes from formal and informal education [29]. assert that university students, who stand for the future as a vibrant sector of society, need to be highly financially literate. A crucial skill that may be acquired through educational programs in schools is financial literacy. The results of the study demonstrate that students majoring in administrative sciences and economics have a high degree of financial literacy.

#### 2.3.3 Financial behavior

Financial behavior is measured in terms of materialism, compulsive buying, and propensity to indebtedness. These constructs were widely used in recent research as a measure of FB (financial behavior). The study of financial management behavior examines the actions and decisions made by individuals and organizations about their financial resources and assets. This field of research focuses on understanding how individuals. Financial behavior can be influenced by the alarming financial distress among different industries like banking [31].

The comprehension of saving, investing, and debt decisions can provide insights into the commonalities and differences observed across individuals worldwide. The concept of FB is intricately connected to both FL and financial competence, as shown by Ref. [32]. According to Ref. [33], The ability of a person to successfully manage and exercise control over their financial resources is referred to as financial competence. To evaluate an individual's financial aptitude, researchers employ two distinct types of measures. The first type pertains to financial behavior measurements, as discussed by Ref. [34]. The second type is based on a combination of behavior and result measures, as explored by Ref. [33]. [32] have identified five distinct categories of financial competence, namely: the management of funds to satisfy basic needs, effective cost management, proactive financial planning, educated decision-making on financial products, and remaining up-to-date with relevant financial information. The lower ratings of young individuals in both objective and subjective financial literacy raise concerns about their money management behavior [35], The financial competence index and perceived financial capability in the 18–24 age range [32]. [36] have offered empirical proof in favor of the hypothesis that financial management practices and financial education are related. According to their findings, those who are better educated about money generally behave better when it comes to money, as seen by higher scores on pertinent metrics. In the past, consumer debt has been used as a stand-in indicator to evaluate how well people manage their finances [37]. Additionally, a financial management behavior measure has been used in multiple studies by earlier research [38,39]; however, It is significant to remember that this research have only focused on a restricted range of domains [40]. examines many facets of insurance [35]. categorized financial behavior characteristics as follows: worry, interest in financial matters, intuitive decision-making, the need for precautionary saves, and discretionary spending [41]. acknowledged the necessity for a psychometrically verified and comprehensive scale, which successfully fulfils the validity and reliability standards. According to the findings of [42]. There is proof that financial management practices follow hierarchical tendencies. According to the survey, 66 percent of the sample managed their cash, 45 percent used credit facilities, 33 percent managed their savings, and 19 percent used successful investing strategies. Most of the study that has been done in the field of finance concentrates on people who are usually referred to as "rational agents" and who have the intention of maximizing their utility [43]. Nevertheless, there are variations among individuals on how they handle their finances. The study conducted by Ref. [39]G confirms the several aspects that impact financial management behavior, including financial knowledge, financial literacy, and internal self-control. As per [44] According to studies done from an Indian perspective, the use of electronic banking services, financial awareness, and help-seeking behavior all have an impact on financial management behavior. According to some academics, there are four psychological elements that influence financial conduct. These characteristics are overconfidence, excessive optimism, danger, and herd behavior [45]. Additionally, the influence of product knowledge and product engagement has been identified as significant [46].

#### 2.3.4 Relationships: financial literacy, materialism, and compulsive buying behavior

According to Ref. [47], financial management techniques can specifically influence materialism and compulsive purchasing behavior [48]. emphasize that training in Proficiency in money management can effectively mitigate and address compulsive buying tendencies. since financial management practices play an important role in this behavior. According to Ref. [49], those with less financial literacy have a higher probability of overestimate the price of credit, which makes them more prone to debt. Additionally, those with poor financial literacy may find it more difficult to amass wealth because they are more prone to take on debt when they are still young [50].

Materialism is the concept of assigning value to physical possessions and the excessive priority placed on their acquisition and ownership [51]: [52]. Individuals demonstrate materialistic values when they prioritize the purchase and ownership of material possessions, considering materialism as a measure of success and a crucial factor in attaining pleasure [38]. The concept of social value is commonly seen in established Western countries, such as the United States [53], as well as in developing markets like China [54], where it has been associated with the adoption of Western consumer lifestyles [55]. While previous studies have traditionally seen materialism as an inherent characteristic of individuals [51], our research takes a more current perspective by considering materialism as a socially acquired value that may influence a whole community.

The direct and indirect impacts that permeate these relationships mean that these influences are not separated from one another [56]. Given that the effects of materialism are connected, materialistic conduct specifically influences compulsive buying behavior.

The inclination to debt, on the other hand, is directly impacted by obsessive purchasing behavior. When consumers lose their ability to regulate their purchases [57],discovered that they will likely acquire products they won't use, are more than they need, or are more expensive than they can afford, which might cause financial difficulties.

According to Ref. [58], Financial literacy can have a substantial impact on preventing obsessive purchase behaviors and countering materialistic values in this context. Additionally, financial literacy is made to achieve just that, as per [59], because it is significantly more advantageous to mitigate consumers' credit issues than to try to repair them later. Financial education has recently increased because of rising loan acquisition and credit card loan delinquency rates. Financial knowledge is crucial in influencing responsible financial behavior, as mentioned earlier.

### 2.4 Financial literacy and materialism: hypothesis development

Furthermore, Materialism strongly correlates with one's ability to manage money, and a comprehensive grasp of FL is necessary, according to Ref. [60].

Beyond learning how to manage money, financial literacy also entails having a solid awareness of the ways that materialism and cultural values affect people's lives.

This awareness is essential for individuals to be considered financially educated. Particularly for young people who are constantly surrounded by materialistic messages from social media and commercials that suggest popularity, contentment, and physical attractiveness can be obtained by material possessions [60]. Highlight the need for financial literacy training. Additionally, those who are materialistic may feel the "pain of knowing" about their assets because effective financial management might reveal the horrifying effects of their spending habits [61]. Therefore, it can be safely hypothesized that.

H1 Materialism is badly impacted by financial literacy.

#### 2.4.1 Financial literacy and financial debts

[49] Highlighting this perspective by noting that individuals with limited financial literacy frequently underestimate the expenses associated with credit, leading to a higher likelihood of accumulating debt. As a result, families with lower financial literacy typically assume higher levels of debt and may even become over-indebted. Due to their higher propensity for accumulating debt at an early age, those with inadequate financial literacy may encounter challenges in saving money and accumulating wealth [62].

We also have indirect impacts to add to this notion. Because financial literacy promotes the development of stronger money management skills, which in turn enable people or households to maintain control over their income and expenditure. These behaviors encompass activities such as establishing a financial plan, ensuring timely bill payments, practicing frugality, effectively handling credit card debt, and maintaining awareness of one's overall financial assets and liabilities [63].

In this regard [49], shown that those who incur large expenditures for debt are typically less knowledgeable about finances and are also more inclined to accumulate debt.

They did this by evaluating the correlation between FL and consumer credit. Thus, it is postulated that materialism and compulsive purchasing behaviour would contribute to the indirect connection between financial literacy and the inclination towards debt. Based on these factors, we have the following relationship: the lower the inclination to debt, the better the level of financial literacy. Therefore, it can be concluded as.

H2 Financial literacy influences negatively a propensity to debt.

#### 2.4.2 Financial literacy and compulsive buying behavior

Financial management techniques like these learned through financial education, are advised as a strategy to stop overspending, and compulsive shoppers are likely unaware of or not using these techniques. Big spenders are more likely to be highly materialistic and to engage in ineffective money management techniques [47]. It is worth mentioning that those who possess strong financial management abilities serve as signs of preserving wealth. Consequently, their actions can moderate the connection between materialism and morality.

Consequently, certain researchers have suggested that providing financial instruction to individuals with a tendency to shop excessively could improve their ability to handle money and effectively put an end to the pattern of compulsive shopping and the resulting accumulation of debt [64].

According to a [59] study, materialism may encourage excessive use of credit and, as a result, contribute to debt by influencing attitudes toward borrowing money. This occurs because materialistic people are more inclined than others to think that borrowing money for certain types of purchases is suitable and acceptable. This led the author to conclude that altering one's attitude toward money would be another way to reduce one's propensity to debt and that this would fall under the category of financial literacy.

Financial management techniques, according to Ref. [58], it has been found that such variables significantly anticipate compulsive behavior following the regulation of materialism, and mitigate the effect of materialistic ideals on incontrollable behavior. These findings endorse the presence of funds management approaches, which may be achieved through financial literacy, in existing psychological therapies. They also show that people with poor money management skills and a strong materialistic mindset are more likely to struggle with compulsive shopping.

Confirming this evidence [61], found that, after accounting for various socio-demographic parameters, effective money management is significantly correlated with higher savings rates and inversely correlated with compulsive buying. Management, budgeting, and saving lessons might be included in secondary school practical teaching units to help students avoid compulsive shopping behaviors [58]. Financial education has been backed by studies, and it is anticipated that these programs may affect compulsive buying [65]. Therefore, it can be hypothesized that.

H3 Compulsive shopping is negatively impacted by financial literacy.

### 2.5 Conceptual model of research

The proposed model, which is depicted in Exhibit 01, was built by combining the constructs and hypotheses as previously described.

#### 2.5.1 Recent research

There are various types of studies conducted on financial literacy. With more than 4000 students [66], assesses the level of financial education of students in public high schools based on individual, demographic, and social characteristics. Another study by Ref. [66] examined the efficacy of the theory of TBP in forecasting credit card debt and student loans among undergraduates from Brazil and the United States of America, as well as their stated financial well-being. The following paragraphs provide a quick overview of the numerous earlier studies that have been conducted so far.

The study by Ref. [50] claims that social media platforms act as a source of information, which might explain inequalities in literacy levels. Financial markets necessitate well-informed and logical investors who possess a good knowledge of financial markets.

The usage of social media and social networks has transformed academics, government officials, and financial professionals into suppliers of financial knowledge.

In addition, followers of social media users may find material from their friends, official media sources, charitable pages, and accounts. The general public lacks advanced financial understanding, making it difficult for them to understand specific pieces of information or correctly interpret media reports [30].

Furthermore [67], Employed structural equation modelling (SEM) to investigate factors influencing the financial wellness of female students (n = 764) residing in either So Paulo or New York, with a specific emphasis on the association with their credit card usage patterns [68]. analyzed undergrads from the city of So Paulo and discovered relationships between personality traits and routines involving risky financial conduct when using credit cards. The study by Ref. [69] Creates and evaluates models for evaluating the financial literacy of college students.

Therefore, this research suggests that financial literacy is a complicated, multifaceted phenomenon that influences other behavioral characteristics directly. However, no research has been done to demonstrate the combined benefits that financial literacy can have on someone who engages in compulsive shopping and materialistic behaviors, or how much this affects their tendency to be in debt. Consequently, we may comprehensively evaluate the characteristics of financial literacy in relation to these behavioural elements by creating and validating a model that measures the direct impact of financial literacy around materialism and obsessive buying, and tendency to debt.

## 3 Profile of respondents and research methodology

Omani citizens made up the study's target demographic, and by the time the data-collecting period ended (in Nov 2022), 233 valid instruments had been collected. The survey was performed haphazardly, outside, by reaching out to students who were willing to participate. The data collecting tool (Table: 5) included 5-point Likert-style questions to assess respondents' propensity for debt, materialism, and compulsive buying as well as multiple-choice questions to assess respondents' profile. The six factors used to measure the study's key theme i.e. financial literacy, utilising a tool derived from research conducted by Parrotta and his colleagues.

### 3.1 Respondents' profile

Table no 1 represents that, 57.3 percent of the sample of 233 people were women, 75.4 percent were single, 20.8 % of people were married, 55.3 % were childless, and the average age was 30. Regarding education levels, 71.2 percent of high school graduates and 14.1 percent finished higher education, 10 % of the respondents held a postgraduate degree as their greatest level of education. The majority of the parents' highest level of education was primary school (52.7 percent). The vast majority of 65.5 percent of respondents had a monthly salary of up to OR 1200. Firstly, the goal of the article is to validate the components utilized by FCA once we know the profile of the respondents.

A factor based on the mean punctuation of two groups of MCQs from studies by Refs. [50,70], and the NFCS, 2013 was created to assess the level of financial understanding. The first section, titled "Basic Knowledge," consists of five questions and is intended to test a person's intellect of basic financial concepts such as inflation, tax rates, and the worth of money over time. Five questions make up the second group (advanced knowledge), which examines the level of education about complicated financial tools.

Then, there are scale questions that assess indebtedness inclination, such as [71] developed this idea. The scale consists of 17 questions that are meant to identify people's financial habits, how they approach purchases, and others among other things, if they think it is sufficient to buy in the future. The study employed a [72] nine-item scale to identify materialism. That is the more the person agrees, the more materialistic he/she is.

In keeping with this viewpoint, our goal was to comprehend the individuals' compulsive behaviors and purchasing habits. To do this, we used a scale that [73] modified and reapplied in Oman. This scale has seven questions that helps in visualizing how respondents are consumerism-oriented, or how they act toward the variety of goods and services offered, such as whether they only purchase when necessary or purchase for the pure pleasure of consumption. Consequently, the higher the level of agreement among respondents with the supplied information, the greater their tendency to engage in compulsive purchase behaviour.

## 4 Analysis and interpretation

### 4.1 Scheme of analysis

With the aid of SPSS 20.0® and Lisrel 9.00 software, CFA and SEM were used to examine the data following a suggestion by Ref. [74]. However, suggestions are for values fewer than five for the x 2/df. The values for CFI, GFI, NFI, and TLI are advised to be larger than 0.95, whereas the values for RMSR and RMSA are advised to be fewer than 0.08 and 0.06, respectively [75].

### 4.2 Assessment of unidimensionality, reliability, and validity

A statistical test was performed to ascertain the significance of the factor loads. The magnitude of the factorial load must also be observed in CFA, and variables with low loadings should be eliminated from the model. Cronbach's alpha [76] was used to check the construct's reliability, with values greater than 0.7 being acceptable. By analyzing standardized residuals, it is possible to confirm the construct's uni-dimensionality. The results for CFA are shown in Exhibit 01.

Following the components' validation, an indicator was developed to gauge each person's understanding of basic knowledge of finance. In order to achieve this objective, we adhered to the recommendations made by Ref. [69], who created a methodology to determine the degree of financial literacy by weighing the measurement criteria and developing a first-level construct with three constructs, each of which represented a component of financial literacy and financial behavior (see Table 1).

Table 2 explains the fit indices of the scale of constructs used in the present article. The initial purpose of CFA was to assess the constructs by verifying their convergent validity, unidimensionality, and construct reliability. Convergent validity was also established through an independent *t*-test. The values for all the scales were above the recommended 1.96. The results are shown in Exhibit 02.

**Table No 1:** Respondent profile- age, gender, and income.

Qualification	Frequency	Percent
Undergraduates	17	7.2
Graduates	145	62.2
Masters	71	8.12
**Total**	**233**	**100**
**Gender**	**Frequency**	**%**
Male	145	62.2
Female	55	38.8
**Total**	**233**	**100**
**Marital status**	**Frequency**	**%**
Single	178	76.39
Married	55	23.06
**Total**	**233**	**100**
**Income (monthly)**	**Frequency**	**%**
>500 OR	145	62.2
>1000 OR	38	16.3
<1500 OR	18	22.4
**Total**	**233**	**100.0**

**Table 2:** Fit indices for the study scales.

Scale	Scale Version*	GFI	NFI	NNFI	CFI
**FL**	Original	0.68	0.53	0.50	0.58
	Refined	0.90	0.80	0.70	0.80
**ML**	Original	0.95	0.96	0.90	0.97
**CB**	Original	0.85	0.80	0.78	0.81
**TD**	Original	0.90	0.94	0.74	0.93

Table 3, explains the reliability of the study scales that have been used for the study. The overall reliability seems to fit according to the Cronbach alpha.

**Table 3:** Scale reliability of the study scales.

CONSTRUCT	CRONBACH ALPHA	CONSTRUCT RELIABILITY	VARIANCE EXTRACTED
**FL**	0.77	0.79	0.46
**ML**	0.84	0.96	0.80
**CB**	0.88	0.89	0.67
**TD**	0.85	0.85	0.55

### 4.3 Examining the control variables

For the current study, four characteristics of the respondents were included as control variables, including *age*, marital status, income, and *gender*.

In order to determine the necessity of accounting for the impact of these variables in the structural analysis, all of the control variables were incorporated into a correlation matrix alongside the study's constructs. The results are given in Table 4.

**Table 4:** Correlation matrix for control variables.

Control variable	Test	FL	ML	CB	TD
Age	Pearson Correlation	−0.30	−0.13	−0.14	−0.11
	Sig. (2-tailed)	0.00	0.21	0.18	0.30
Gen	Pearson Correlation	0.27	0.05	0.08	0.02
	Sig. (2-tailed)	0.01	0.60	0.41	0.79
MS	Pearson Correlation	0.25	0.36	0.12	0.15
	Sig. (2-tailed)	0.01	0.00	0.25	0.15
Income	Pearson Correlation	0.28	0.05	0.09	0.03

Table 5 depicts that there is no significant relation among the control variables and the research variables. In other words, there is a weak. Therefore, it is found that the hypothesized control factors have no substantial impact on the associations, and as a result, all of them were excluded from the model.

**Table 5:** Constructs and retained items after scale purification.

Constructs	Description of Items
Financial Literacy: Independent Variable	
V1	Establishing a consistent routine of saving and adhering to it is crucial for a family**.
V2	It is important for families to have explicit financial objectives that aid in determining spending priorities**.
V3	A defined budget is crucial for effective financial administration**.
V4	As long as one consistently meets the monthly payments, there is no need to worry about the length of time involved.
V5	I do the practice of recording and managing my expenditures.
V6	I do price comparisons when making purchases.
**Tendency To Debt: Dependent Variable**	
V7	Acquiring a loan is beneficial as it enables one to have a fulfilling lifestyle**.
V8	It is advisable to make a purchase today and defer the payment for a later time**.
V9	Credit is an essential element of contemporary living.
V10	It is more beneficial to incur debt than to deprive children of Christmas presents**.
V11	Taking out a loan can be advantageous in certain circumstances**.
V12	I have a propensity for taking risks with my finances.
**Materialism: Dependent Variable**	
V13	Everything that I possess serves as a major indicator of my level of success in life.
V14	I take satisfaction from having items that attract admiration from others**.
V15	I hold great admiration for individuals who possess luxurious residences, automobiles, and attire**.
V16	Purchasing something brings me great satisfaction.**.
V27	I greatly appreciate indulging in luxurious experiences in my life**.
V18	Acquiring specific possessions that I currently lack would significantly improve the quality of my existence**.
**Compulsive Buying Behavior: Dependent Variable**	
V19	If there is any extra money remaining after the month, I am compelled to use them towards expenditure**.
V20	I think my purchases may cause shock among others if they were aware of them.
V21	I make purchases even when I lack the financial means to do so**.
V22	I knowingly issue checks while having inadequate money in my bank account to cover them**.
V23	I purchase items in order to enhance my personal well-being.
V24	I get sensations of anxiety when I spend a day without making a purchase**.

### 4.4 Structural model

The model was subsequently constructed and examined using Structural Equation Modelling (SEM), with the statistical significance of the projected regression coefficients being confirmed to evaluate the suggested theoretical framework and the model's refined indices. The refined indices used to validate the measurement model were the same as those used before.

The findings indicate that the model, the incorporation of every factor from the initial scale for each of the 4 constructs is insufficient due to the x 2/df ratio exceeding five and some fit indicators exceeding the minimum limits. Thus, to identify appropriate models, two primary approaches were employed: the removal of variables that were not statistically significant and the inclusion of correlations between errors of variables.

The standardized coefficients for each construct, along with the chi-square statistics (x2), root mean square residual (RMSR), root mean square error of approximation (RMSEA), goodness-of-fit index (GFI), comparative fit indices (CFI), normed fit index (NFI), and Tucker-Lewis Index, were utilized to assess the convergent validity (TLI) of each factor. The literature contains diverse opinions of acceptable values.

After implementing these changes, both models demonstrated satisfactory levels of convergent validity, as indicated by the CFI, GFI, NFI, and TLI indices beyond 0.95, and the RMSR and RMSEA indices below 0.08. Additionally, the models exhibited reliable results, with reliabilities surpassing 0.7, and demonstrated uni-dimensionality, as found by all standardised residuals being below 2.58. After evaluating the final structures, it is clear that Six out of the fifteen factors created for financial attitude were kept.

The variables that have the greatest influence on this feature are those that investigate whether respondents consider a documented budget and a contingency plan for potential loss of income for any family member to be crucial for good financial management.

The variables that have the most major influence on an individual's successful financial performance are their regular practice of budgeting their expenses and setting aside a portion of their monthly income to address emergencies or shortfalls. Six of the nine suggested characteristics were used to construct the materialism model, and individuals who supported the notion of having more material possessions are more likely to exhibit materialistic behavior. Regarding compulsive buying, it was observed that five out of the seven questions were included in the final structure/model. Individuals who endorsed the concept of purchasing items despite being aware of their inability to afford them demonstrated the greatest inclination towards engaging in this behavior.

The scale's most unstable component was propensity to indebtedness, as only six of the original 17 variables were included in the final model, and individuals who are more likely to obtain loans are those who are more propensities to becoming indebted.

## 5 Findings and conclusion

### 5.1 Findings

Financial literacy shows a detrimental impact on materialism and compulsive buying behavior, according to the two latest direct links that were postulated (H1 and H3). Financial literacy is a reliable indicator of people having better compulsive buying behaviors, as evidenced by the influence of materialism coefficient of 0.88 and the greatest coefficient (0.80) of any relationship among the initially proposed relationships between compulsive buying and financial literacy. This provides evidence for the argument that it is necessary to create and include practical programmes into secondary education curricula in order to combat compulsive buying behaviours. These programmes should focus on promoting financial literacy and teaching essential personal financial skills. As suggested by Ref. [77] awareness is a crucial aspect of financial literacy when comes to financial decision-making.

Financial literacy showed a direct effect on indebtedness propensity, supporting hypothesis 2 (H2). In particular, when it comes to financial literacy, these findings support the OECD's (2015) research, which found that financial conduct, the second aspect of financial literacy, was crucial and unquestionably the most significant of the three because it determines whether financial balance or imbalance exists. Additionally [78], noted that Merely possessing financial attitudes and information is inadequate for achieving financial stability. In addition to comprehending the principles and being ready to take action, it is crucial to also have an awareness of one's own financial condition.

Two assumptions were confirmed about the indirect impacts in the constructs, when all of the impact that financial literacy has on the behavioral traits were considered, it became clear that materialism, propensity to borrow money, and compulsive buying were the three behavioral elements that financial literacy had the largest influence on.

### 5.2 Conclusions

These findings are crucial for understanding the value of financial literacy that has a broader scope than just assisting individuals with their finances to other behavioral issues that have an impact on a society's ability to manage its finances. According to Ref. [62], group cognitive-behavioral therapies that taught participants how to manage their money and other techniques employed by people who are deemed financially intellect effectively decreased compulsive buying and, as a result, debt.

The report titled "International Survey of Adult Financial Literacy Competencies" in December 2022 has increased the importance of the current research. The report outlines the results of the research that examined the financial understanding, financial attitude, and financial behavior of adults between a range of 18 and 79 from 30 different nations. Notably, this study includes data from Oman for the first time.

Despite the global low level of financial literacy, our rating was significantly more diminished, falling 1.2 % points less than the global average (OECD, 2016). Therefore, it is imperative to promptly and efficiently implement strategies to mitigate the impact of inadequate financial literacy on one's lives and, by extension, on the economic issues.

Financial security for both an individual and the economy as a whole now heavily depends on financial literacy [79], it could also improve the overall performance of the portfolio. Nevertheless, studies have demonstrated that financial literacy is a multifaceted phenomenon that influences various behavioural characteristics. The objective of this study is to create and verify a structural equation model that explores the relationship between financial literacy, materialism, compulsive buying, and predisposition to debt. The model allows for the identification of the collective influence of financial literacy on these variables.

The proposed constructs were evaluated because there were no unified structures. Consequently, out of the total of 61 questions, 36 were confirmed to be accurate, indicating a certain degree of inconsistency in the scoring scales. The structural model and three hypotheses that showed Insufficient calibration indicators in the original model and required some revisions were then combined in an integrated model that was created. Some relationships between the mistakes were included in these adjustments.

The most significant direct association among the supported hypotheses was between financial literacy and Materialism, demonstrating that financial literacy has a beneficial impact on people and enhances materialism.

Finally, it was discovered that when looking at the overall effects of financial literacy on the behaviors examined, compulsive buying behavior had a significant impact, after materialism and then propensity for debt.

Given the foregoing, this study is exceptional and cutting-edge in that it assesses the real direct effects of financial literacy on other behavioral characteristics that have previously been examined separately. According to the study, financial literacy has far greater effects than past academic studies have suggested because, from an academic standpoint, the main emphasis up to this point has been on detecting only its impact on other behaviors.

### 5.3 Implications

These findings are crucial for the creation of public policies and for other agents recognizing that the benefits of financial literacy extend beyond the direct effects it has on those who possess it and how it may benefit those in need even more broadly. Individuals experience additional psychological symptoms, including obsessive shopping. Moreover, it can assist in the development of treatments for those with materialistic and obsessive shopping tendencies, as well as those who exhibit a strong propensity for debt. Additionally, financial institutions could have the chance to apply this knowledge to better comprehend their consumers' financial literacy and create items that would suit each client's needs. The study plays a vital role in determining the relationship between financial literacy and the behavioral aspect of investment, the originality lies in the respondent of the study which is students from Generation Z [80]. explored compulsive buying among Indian consumers. Our study supports the findings of this paper, furthermore [77] behavioral dynamics of volatile assets like crypto-currency are considered in this article. Financial literacy plays an impeccable role in deciding investment opportunities [81]. financial literacy plays very important role in the Islamic finance and this research shows importance and need of financial literacy in Oman. One of implications of this research could be that, financial distress among different industries could also be avoided.

## Availability of data and material

Not applicable.

## CRediT authorship contribution statement

**Mohammad Shahfaraz Khan:** Methodology, Conceptualization. **Imran Azad:** Validation, Investigation. **Sanyo Moosa:** Supervision, Project administration, Formal analysis. **Mohd Yousuf Javed:** Writing – review & editing, Validation, Methodology.

## Declaration of competing interest

The authors declare that they have no known competing financial interests or personal relationships that could have appeared to influence the work reported in this paper.
